# Indigenous solutions to the climate and biodiversity crises: A reflection on UNDRIP

**DOI:** 10.1371/journal.pgph.0002060

**Published:** 2023-06-14

**Authors:** Nicole Redvers, Yuria Celidwen, Quanah Yellow Cloud, Anpotowin Jensen, Cicilia Githaiga

**Affiliations:** 1 Schulich School of Medicine & Dentistry, University of Western Ontario, London, Ontario, Canada; 2 Arctic Indigenous Wellness Foundation, Yellowknife, Northwest Territories, Canada; 3 Othering & Belonging Institute, University of California, Berkeley, Berkeley, California, United States of America; 4 School of Social Work, University of New England, Biddeford, Maine, United States of America; 5 School of Civil and Environmental Engineering, Stanford University, Stanford, California, United States of America; 6 Wangari Githaiga & Co Advocates, Nairobi, Kenya; PLOS: Public Library of Science, UNITED STATES

*“Indigenous Peoples have the right to maintain and strengthen their distinctive spiritual relationship with their traditionally owned or otherwise occupied and used lands*, *territories*, *waters and coastal seas and other resources and to uphold their responsibilities to future generations in this regard”* [1].

(Article 25, UNDRIP)

The United Nations (UN) General Assembly formally passed a resolution (A/76/L.75) “recognizing the right to a clean, healthy, and sustainable environment as a human right” in 2022 [2]. This resolution is similar to resolution 48/13 of the UN Human Rights Council (UNHRC), passed in October 2021, which was said to be the first time the right to a clean, healthy, and sustainable environment was recognized explicitly at the global level for all of humanity. These two resolutions, however, are conspicuously informed by Indigenous Peoples and their stewardship knowledges from around the globe. Fifteen years prior, the United Nations Declaration on the Rights of Indigenous Peoples (UNDRIP) was signed by the UN General Assembly with 144 original signatories (148 Member States currently), which holds specific provisions for Land and Cultural Rights including the ability of Indigenous Peoples to “…uphold their responsibilities to future generations…” [1].

UNDRIP is the most comprehensive international framework on the minimum recommendations for the survival, respect, and dignity of Indigenous autonomy and participation [1]. While the Declaration elaborates on human rights standards as they pertain to the fundamental freedoms and specific situations of Indigenous Peoples worldwide, due to the lack of legally binding enforcement, the Declaration has varying levels of support and advancement within each Member State towards full implementation. Among the fundamental rights elevated by UNDRIP are specific provisions for Indigenous self-determination in economic, social, and cultural development (*preamble*, *Articles 3*, *4*); ‘free, prior, and informed consent ‘(FPIC) (*Articles 10*, *11*.*2*, *19*, *28*, *29*.*2*, *32*.*2*); and the right to Indigenous Lands and Territories (*Articles 10*, *24*.*1*, *25*, *26*, *27*) [1].

The process to establishing UNDRIP was long and strenuous. One of the first Indigenous leaders to push for the recognition of Indigenous sovereignty at a global institutional body was Chief Deskaheh, a Haudenosaunee hereditary chief. In 1923, Chief Deskaheh was denied the ability to speak, and was prohibited from being given a platform to address the assembly of the United Nations, when it was still known as the ‘League of Nations’ [3]. The year 2023 is the 100th anniversary of Chief Deskaheh’s push for Indigenous rights at the international level [3]. Over eight decades later UNDRIP was ultimately passed [1]. UNDRIP today is an arguably underutilized instrument at local, national, and international levels for addressing our converging global crises—including the climate and biodiversity crises.

International climate and biodiversity negotiations have continued to marginalize Indigenous Peoples from direct participation, outside of observer statuses, in the decision-making processes to address the climate and biodiversity emergencies. This despite the foundational tenants of UNDRIP and Indigenous sovereignty acknowledging Indigenous Peoples as a self-determined Peoples. Indigenous Peoples are the most effective in conserving and stewarding the remaining eighty percent of the world’s remaining biodiversity, which includes stewarding the largest remaining carbon sinks on the planet (equivalent to 33 times the global energy emissions of 2017) [4]. Increasing evidence clearly demonstrates that Indigenous Peoples’ traditional land management strategies are noticeably more effective in climate mitigation, especially in those areas where communities own the legal rights of governance over their Ancestral territories. The Food and Agriculture Organization (FAO) found that between 2003 and 2016, Indigenous territories, which cover twenty-eight percent of the Amazon Basin, made up only 2.6 percent of its carbon footprint [5]. Latin American countries with the highest percentages of Indigenous populations—like Bolivia, Brazil, Colombia, and Peru—show deforestation rates three times lower in those areas that are owned by community trusts [6]. It may be inevitable to conclude that Indigenous Peoples through their environmental stewardship, have managed to shield all peoples and Mother Earth from the complete collapse of all other ecosystems thus far.

It important to highlight, however, that Land dispossession, extractivist economies, and direct violence against Indigenous Land defenders—including in the name of green economies for climate action—have led to significant pressures on Indigenous Peoples’ abilities to continue their important planetary stewardship roles [7]. Additionally, the push for nature-based initiatives connected to the Convention on Biological Diversity’s goal of protecting 30% of the Earth’s lands, oceans, coastal areas, and inlands waters, have been a concern for many Indigenous communities. Indigenous advocates are concerned that the 30x30 biodiversity agenda risks millions of people being evicted from their ancestral territories if implemented poorly given the long standing history of conservation violence against Indigenous Peoples around the globe [8]. Indigenous Land and cultural rights through mechanisms such as UNDRIP cannot be separated from international climate and biodiversity agendas.

In this Opinion we therefore firmly premise—as Indigenous Peoples have been “declaring for decades”—that climate change and biodiversity loss are directly caused by colonialism [9] and the perpetuation of colonial practices, strategies, policies, and judicial systems. In its sixth report summary, the Intergovernmental Panel on Climate Change (IPCC), unequivocally states that “[p]resent development challenges causing high vulnerability are influenced by historical and ongoing patterns of inequity such as colonialism…(*high confidence*)” [10]. With this, UNDRIP and other Indigenous-related mechanisms aimed at countering colonialism are put forward as climate and biodiversity solutions-focused frameworks for the re-establishment of authority of Indigenous Natural or First Law [11]; the recognition of Ancestral legal personhood designations (e.g., Rights of Nature) as common under Indigenous Natural Law [12]; and clear provisions for Indigenous governance and Land tenure rights. We additionally push for the universal recognition of UNDRIP regardless of which Nation States are signatories. This call is particularly important now that the Kunming- Montreal Global Biodiversity Framework (The GBF) “… acknowledges the important roles and contributions of Indigenous Peoples and local communities as custodians of biodiversity and as partners in its conservation, restoration and sustainable use” [13].

Further inquiry is needed, however, to further distil the current UNDRIP framework within the multitude of established legal Treaties that exists between Nation States and individual Indigenous Nations in some countries around the globe. For example, the 1851 and 1968 Fort Laramie Treaties concluded between the Great Sioux Nation or Oceti Sakowin and the United States (US) remain legally binding till this day according to the US Constitution and Miguel Alfonso Martinez’s 1999 UN Study on Treaties and Agreements. Individual Nation to Nation Indigenous Treaties are in many cases seen to supersede international agreements which is honoured through the individual provisions within UNDRIP; however, individual Indigenous Nations with Treaties may have additional context to add to this dialogue in the context of Indigenous solutions to the climate and biodiversity crises which we hope to see further platformed [14]. Nonetheless, UNDRIP should be further platformed within current and ongoing climate and biodiversity negotiations at multiple scales of influence and across all sectors and governance.

*“Working in alliance with nature and her Natural Laws is the key to ensuring our survival…The reason we have climate change is because we have broken Natural Law…Natural Laws are innate to all living beings*. *They are the invisible laws that govern all life*. *All living beings*, *including Mother Earth herself*, *are governed by Natural Laws–whether they know it or not”* [15].

~Elder David Couchere (Nii Gaani Aki Innini) of the Anishinaabe Nation.
